# Integrating Exposure Assessment and Process Hazard Analysis: The Nano-Enabled 3D Printing Filament Extrusion Case

**DOI:** 10.3390/polym15132836

**Published:** 2023-06-27

**Authors:** Stratos Saliakas, Spyridon Damilos, Melpo Karamitrou, Aikaterini-Flora Trompeta, Tatjana Kosanovic Milickovic, Costas Charitidis, Elias P. Koumoulos

**Affiliations:** 1 Innovation in Research & Engineering Solutions (IRES), 1780 Wemmel, Belgium; esaliakas@innovation-res.eu (S.S.); sdamilos@innovation-res.eu (S.D.); 2 Research Lab of Advanced, Composites, Nanomaterials and Nanotechnology (R-NanoLab), School of Chemical Engineering, National Technical University of Athens, Zographos, 15780 Athens, Greece; mkaramitru@chemeng.ntua.gr (M.K.); ktrompeta@chemeng.ntua.gr (A.-F.T.); tkosanovic@chemeng.ntua.gr (T.K.M.); charitidis@chemeng.ntua.gr (C.C.)

**Keywords:** polymer extrusion, occupational safety, exposure assessment, nanocomposites

## Abstract

Nanoparticles are being used in novel applications of the thermoplastics industry, including automotive parts, the sports industry and leisure and consumer goods, which can be produced nowadays through additive manufacturing. However, there is limited information on the health and safety aspects during the production of these new materials, mainly from recycled sources. This study covers the exposure assessment to nano- and micro-size particles emitted from the nanocomposites during the production of filaments for 3D printing through a compounding and extrusion pilot line using recycled (post-industrial) thermoplastic polyurethane (TPU) and recycled polyamide 12 (PA12), which have been also upcycled through reinforcement with iron oxide nanoparticles (Fe_3_O_4_ NPs), introducing matrix healing properties triggered by induction heating. The assessment protocol included near- and far-field measurements, considering the extruder as the primary emission source, and portable measuring devices for evaluating particulate emissions reaching the inhalable zone of the lab workers. A Failure Modes and Effects Analysis (FMEA) study for the extrusion process line was defined along with a Failure Tree Analysis (FTA) process in which the process deviations, their sources and the relations between them were documented. FTA allowed the identification of events that should take place in parallel (simultaneously) or in series for the failure modes to take place and the respective corrective actions to be proposed (additional to the existing control measures).

## 1. Introduction

Nanocomposites are composite materials having one of the phases with dimension in the nanometre range and offer the prospect for substantial enhancement of material attributes such as thermal, chemical, mechanical, optical, magnetic and electrical properties [1]. Extensive research interest has been dedicated to polymer-matrix nanocomposite materials for at least two decades [1,2], while scientific efforts are still garnered towards the advancement of this field, and extension of nanocomposite material application in fields such as automotive, packaging and biomedicine [3]. The most common materials used as nanofillers in polymer materials are carbon-based nanoparticles such as carbon nanotubes [4,5,6], nano-oxides, e.g., magnetite (Fe_3_O_4_) [7], nanoclays [8] and nano-carbides, such as silicon carbide (SiC) [9]. Conventional processing techniques are widely established in the polymer industry, such as the compounding process that can use nano-enabled feedstock [10]. Additionally, additive manufacturing technologies increasingly use polymeric filaments enhanced with nanoparticles for multifunctionality [4].

Technical challenges within nanocomposite manufacturing include the achievement of proper dispersion and distribution of nanoparticles in the matrix, the avoidance of nanoparticle agglomeration and the optimization of the bond between matrix and filler in the interface layer [1]. Among the various barriers to the broader adoption of nanocomposite materials from the industry are the upscaling considerations [2]. Additionally, nanosafety may be a deterring factor since dedicated approaches for risk assessment and investments in terms of safety infrastructure are required to address potential occupational safety concerns arising from the application of nanomaterials. Nanosafety aspects related to occupational safety during the various life cycle stages of nanocomposite manufacturing (e.g., exposure of workers to released nanomaterials during compounding) may also be examined in the nanocomposite part use phase. Applications of nanocomposites that entail processing (e.g., thermal, mechanical) of the material during its use phase, such as the manufacturing of nanocomposite 3D printing filament, examined in the present study, ought to consider possible nanosafety concerns during the use phase to account for potential emissions during the 3D printing process [11].

Exposure to nano-size and micro-size particles is greatly discussed in the literature due to the potential adverse effects on human health. Nanomaterials present higher toxicity potential than bulk materials of identical chemical composition, due to their smaller size and higher surface area, allowing chemical interactions to occur [12]. Nanomaterials can penetrate the cellular membrane followed by the production of reactive oxygen species (ROS), leading to oxidative stress causing DNA damage, oxidation and denaturation of proteins and enzymes and disruption of mitochondria leading to cell apoptosis as well as greater adverse health effects, such as inflammation, chronic respiratory illnesses and cancer [13]. Nanocomposite processing can lead to the emission of ultrafine (<100 nm) or larger particle sizes throughout the nanocomposites’ life cycle. The ISO/TR 22293:2021 Standard presents in detail the mechanisms of material release from nanocomposite matrices with or without nanomaterials embedded in the matrix [14].

Potential routes of exposure are inhalation, dermal, ingestion and ocular penetration. The respiratory tract is the main exposure pathway to nanomaterials, and particle deposition in the lungs depends on the nanomaterial’s aerodynamic diameter. Particles above >1 μm in aerodynamic diameter tend to deposit in the inhalable zone and can be easily removed, while smaller size particles (<100 nm) tend to deposit in the tracheobronchial and alveolar regions (namely, respirable fraction); thus, they cannot be removed and block the alveoli [15]. Deposition in the alveoli, which are one of the most sensitive parts, can lead to adverse health effects if the particles are not cleared through the immune system [16]. A small fraction of nanoparticles deposited in the alveolar region may be cleared into the bloodstream by absorption and translocate to other organs in the human body [17], while particles that deposit in the respiratory tract can also be cleared to the gastrointestinal tract via the pharynx or to the regional lymph nodes via lymphatic channels [18]. It is worth noting that occupational exposure limits (OELs) are still limited regarding the inhalable and respirable particulate fractions of nanomaterials. Additionally, Larese Filon et al. studied dermal penetration of particles in healthy or injured skin, showing that particle size plays a vital role in exposure assessment in general [19].

The field of nanosafety has displayed rapid research growth within the last decades [20]. Nano-specific risk assessment tools have been developed, often in a web-based application format (e.g., Stoffenmanager Nano [21], SUNDS [22]), which assist in defining occupational risk levels present within a given process/material combination as well as define required controls to mitigate the risk. Various nanosafety-dedicated standards have been developed, including standardized guidelines to occupational safety control definition (ISO/TR 12885:2018) [23], along with guidance on the study of release forms and mechanisms from nanocomposite materials (ISO/TR 22293:2021) [14]. While the most extensively researched subject in nanosafety is the investigation of the inherent hazard and toxicity of the various nanomaterial species, within the spectrum of nanotoxicology [24], the field of nanomaterial exposure assessment, particularly in the occupational setting, has also been speedily evolving [25]. Specialized instrumentation and detailed methodologies for exposure assessment have been adopted by the nanosafety community. Notably, the OECD “Harmonized tiered approach to measure and assess the potential exposure to airborne emissions of MNs and their agglomerates and aggregates at workplaces” [26], presents a reliable and consistent methodology to conduct airborne (nano)particle exposure measurements and analyse the data obtained. The approach has been developed after a systematic study of previously applied techniques in nanomaterial exposure assessment. Technical challenges opposed to performing an accurate exposure assessment still exist, such as the absence of health-based occupational exposure limits (OELs); however, research efforts are being dedicated to ameliorating these issues [27].

It is quite important to note that nanosafety approaches focus on identifying and analysing hazards related to nanomaterial exposures; however, personnel will undoubtedly be exposed to various other occupational hazards within the context of nanocomposite manufacturing. These consist of laboratory or industrial setting safety concerns, such as high temperatures, noise and physical hazards (moving parts and sharp tools). They are investigated and addressed on the basis of a Plan-Do-Check-Act (PDCA) approach, through the principles of the extensively adopted ISO 45001:2018 standard [28].

While the literature is expanding on both occupational safety in polymer manufacturing and nanosafety as distinct fields, research is lacking regarding an integrated approach to evaluate both nano-specific and process hazards. The objective of this work is to conduct a comprehensive risk evaluation for the hazards present within a nanocomposite 3D printing filament manufacturing process through twin-screw extrusion. An airborne (nano)particle exposure investigation through on-site measurements is presented, complemented by safety-oriented Failure Mode and Effects Analysis (FMEA) and Fault Tree Analysis (FTA) studies which elaborate on the various physical hazards present in the manufacturing process.

## 2. Materials and Methods

### 2.1. Nanomaterials and Polymer Matrices

Four different feedstocks were used to produce filaments for Fused Filament Fabrication (FFF) 3D printing through polymer extrusion. The two polymer materials that were studied were post-industrial polymer waste streams that have been mechanically recycled. Specifically, recycled thermoplastic polyurethane (TPU), originated from Elastollan 1164D–TPU (BASF Polyurethane GmbH, Lemförde, Germany), and polyamide (PA), originated from Grilamid L 20A HL NZ natural (Grilamid EMS, Domat/Ems, Switzerland), were used as feedstock materials, to be enhanced with the nanoparticles. Initially, a concentrated masterbatch was prepared through compounding for each case, at a concentration of 10 wt%. with Fe_3_O_4_ nanoparticles (NPs)–Fe_3_O_4_–110 iron oxide nanoparticles 99.5% purity, 20 nm in diameter, 1% PVP-coated, supplied by GetNanoMaterials (Oocap France SAS, Saint-Cannat, France). Afterwards, dilutions with pure recycled polymers resulted in different concentrations, i.e., 2.5, 5, 7.5 and 10 wt%, and 5 m length filaments were produced through extrusion for each concentration. The addition of susceptor materials for induction heating, such as iron oxide nanoparticles, due to their strong magnetic permeability [29], introduce into the thermoplastic nanocomposite functionalities such as induced healing of polymer matrix [30] and on-demand debonding properties for recycling applications [31]. Once exposed to an external alternating magnetic field, MNPs act as the susceptors, converting the energy of the field and generating heat in nanocomposite material by the hysteresis mechanism [32]. Local melting enabled polymer chains’ interdiffusion, which allowed facile bonding/debonding. Several polymer matrices reinforced with Fe_3_O_4_ nanoparticles, including TPU and PA12 under study in the present work, have been investigated for their induced heating and healing ability [33]. All thermoplastic nanocomposites with MNP concentrations from 2.5–10 wt%, resulted in induced heating after a short period of exposure to the alternating magnetic field. The temperature increase was proportional to the MNPs’ concentration, and healing behaviour was observed for the TP nanocomposites that reached a temperature close to the Tm of the matrix (MNPs concentrations 7.5–10 wt%). For changing the material in the compounding system, it is important to effectively clean the extrusion barrel and the screws, to avoid any contamination. The cleaning procedure that was followed included mechanical cleaning on elevated temperatures with a brass brush for the steel parts and chemical cleaning with solvents compatible with the polymers under processing, i.e., tetrahydrofuran (THF) and dimethylformamide (DMF), to remove the TPU traces for processing afterwards for PA12.

### 2.2. Extrusion Pilot Line for 3D Printing Filament Preparation

**Figure 1:**
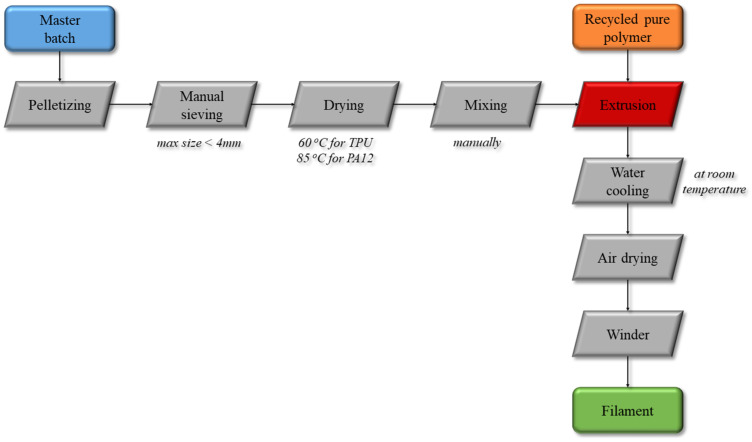
Process flow diagram of the filament production via extrusion.

The production line included all steps from feedstock pre-processing, i.e., pelletizing, sieving, extrusion and filament winding. The process flow diagram is shown below (Figure 1). The masterbatch, initially in rod form, was further pelletized using Collin Teach Line Strand Pelletizer apparatus down to ~4 mm in length, followed by visual quality inspection and manual sieving to ensure that the pellet size is below 4 mm, to be introduced into the extruder feeder hopper. Drying in a standard laboratory furnace for 4 h at 60 °C for the TPU and 85 °C for 12 h for the PA12 was necessary prior to processing. The pellets from the dried nanocomposite masterbatch are mixed with pure polymer matrix pellets and introduced in the volumetric singe-screw feeder for the nanocomposite filament compounding and extrusion process to start, using co-rotating twin-screw extruder Thermo Scientific Process 11 (Thermo Fisher Scientific, Waltham, MA, USA). The polymers were processed in a slightly different temperature range; eight temperature zones cover the barrel length, for a uniform and gradient temperature transition, increasing the melting point of each polymer. For TPU, the range was selected from 105 °C to 210 °C, while for the PA12, the temperature ranges from 180 °C to 225 °C. Once reaching steady state, the produced filament passed through a water-cooling bath at room temperature, followed by a ring blower, that works with compressed air to remove any remaining water from the cooling bath. Prior to winding, a tri-axial laser monitoring system (Zumbach USYS200 and ODAC 13TRIO (Zumbach Electronic AG, Orpund, Switzerland)) was employed to monitor real-time the filament diameter. The rotation speed of the winder (Filament Spooler for Process 11) can be adjusted to set the optimum tension for a constant filament diameter equal to 1.75 ± 0.05 mm (mostly used in additive manufacturing relevant systems worldwide). Some basic information about the workroom and the process are presented in Table 1.

### 2.3. Exposure Measurement Campaign

**Figure 2:**
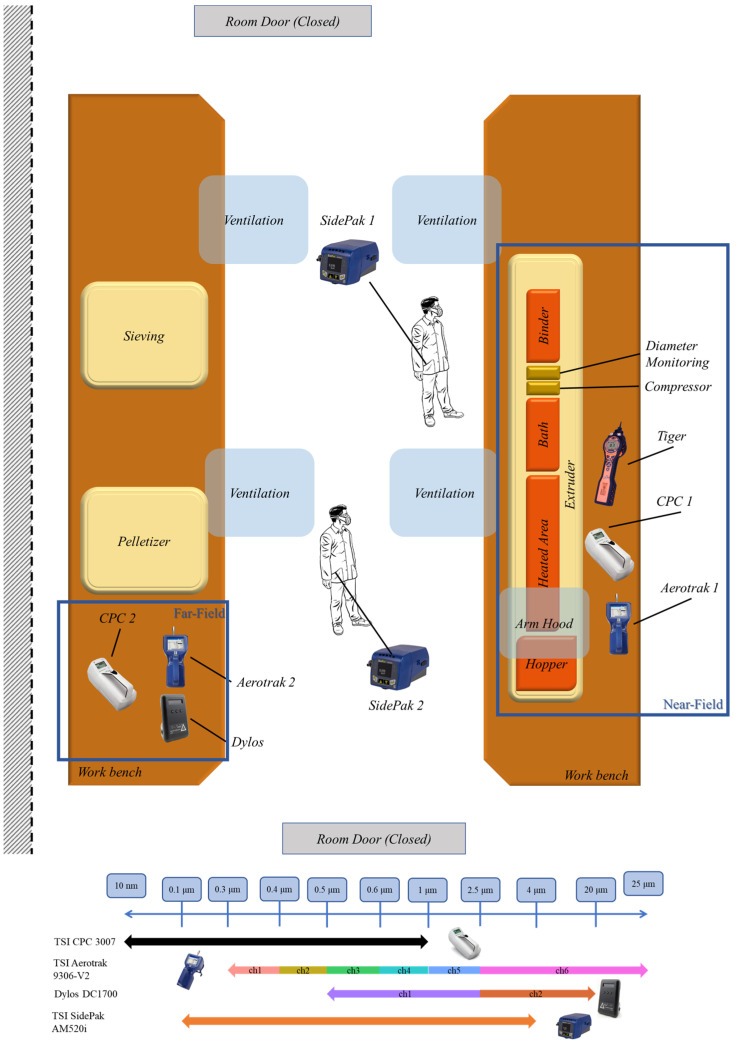
Illustration of workroom and exposure instrument setup and instrument size range detection capabilities. The different measurement channels (chX) are also displayed for each instrument.

An exposure assessment was performed for the evaluation of real-time particle number concentrations in the near- and far-field using condensation particle counters (CPC) (CPC3007, TSC Inc., Shoreview, MN, USA) and optical particle counters (OPC) (Aerotrak 9306-V2, TSC Inc., Shoreview, MN, USA; DC1700, Dylos Corp., Riverside, CA, USA), while personal aerosol monitoring equipment (AM520i, TSI Inc., Shoreview, MN, USA) allowed the evaluation of mass-based concentrations of particles near the breathing zone of the laboratory operators. Two persons were equipped with the portable devices: one laboratory operator and one visitor/observer. Five different exposure measuring equipment types were applied, resulting in a total of eight devices covering near- and far-field emission measurements from the extruder as well as the emissions reaching the breathing zone of the laboratory personnel present. The placement of CPC and OPC instruments in both the near- and far-field enabled the qualitative evaluation of the dispersion and removal of particles emitted due to the process. It also allowed the identification of emission events caused by adjacent activities. The positioning of the instruments is illustrated in Figure 2 along with the extruder line devices and equipment and exposure controls in place within the workplace (mechanical ventilation units and local extraction movable arm hood). The devices with respect to the measurement location and measuring size ranges are shown in Table 2. Figure 2, also, shows the measurement size range channels of Aerotrak and DC1700 instruments to display the sub-micron size range with detail. Additionally, total volatile organic compound (TVOCs) concentration measurements were performed near the extruder using the portable photoionization detector device Tiger (Ion Science Ltd., Royston, UK), measuring TVOCs as isobutylene equivalent in a range of 0–20,000 ppm with 1 ppb accuracy. Time logging allowed the matching of the particle and VOCs emissions with the different tasks performed during the procedure as well as the time recording of the background particle and VOCs concentrations before and after the process operation.

### 2.4. Risk Assessment Methodology

#### 2.4.1. Failure Mode and Effect Analysis (FMEA)

Failure Modes and Effect Analysis (FMEA) methodology was used for hazard analysis and risk assessment in the extrusion pilot line, as described in SAE J 1739-2009 Standard [34]. Through FMEA, potential failure modes of the process components were identified and analysed with their causes and respective health effects and causes, while allowing prioritisation of the corrective actions. Three nodes were identified in the current process presented in Section 2.2 (Extruder, Pelletizer and Blow Dryer), resulting in eight potential failure modes. Following our previous work on similar systems [35] and previous literature studies [36,37], the scope of the analysis was focused on the health and safety of the process line, analysing the potential adverse health effects of each failure mode. Hence, this analysis includes the process steps, the potential failure that can emerge (potential failure mode), the harmful effect on the operator, the potential causes of failure to take place as well as the current process control and recommended actions that can mitigate the risk. FMEA study was conducted by two process operators of the pilot line and four safety consultants; discussion and analysis of existing equipment and potential failures was followed by several walkthrough sessions observing the equipment and all processes within the extrusion pilot line during operation and idle.

Risk prioritisation was based on the calculation of the Risk Priority Number (RPN) as the product of the three elements (severity, detectability and occurrence) of each potential failure mode (shown in Table 3):

- Severity (S) was ranked from one to five, describing the effect per failure mode (one: least severe, five: most severe).
- Occurrence (O) was ranked from one to five and was associated with the likelihood of each failure mode to take place (one: low probability, five: high probability).
- Detection (D) was ranked from one to five and was associated with the difficulty of a failure mode being detected and prevented (one: easy to be detected, five: hard to be detected).

The higher the RPN, the higher the associated risk of the studied mode and the higher is the priority in taking action (Table 4). In this analysis, the risk level may vary from very low (RPN between zero and one) and low (RPN between two and eight), meaning negligible or discretionary remedial actions to be considered, up to very high (RPN between 65 to 125), indicating that operation is not permissible and immediate control actions (i.e., engineering or administrative controls) are necessary.

#### 2.4.2. Fault Tree Analysis (FTA)

A qualitative Failure Tree Analysis (FTA) was conducted—as a supportive study to the FMEA analysis—to analyse the relationship between a failure event during a process and the potential causes in the extrusion pilot line without focusing on their likelihood of occurrence (as described in IEC 61025:2006 Standard). Integrating the FMEA study with FTA analysis has been examined to enhance comprehension of the relation between the events that lead to the investigated failure modes [38]. The FTA assessment comprises of three key elements:

- The top event, which includes the description of the critical system;
- The basic events, which include the low-level identified causes;
- The logic gates (AND, OR), which display the logic connection between the basic events and the top event.

Failure Tree Analysis is based on Boolean logic, where the events are arranged in sequences of series relationships (the “ORs”) or parallel relationships (the “ANDs”). Results for each event are presented in a tree-like diagram using logic symbols to show dependencies among events. In other words, the FTA assessment concerns the identification and analysis of conditions and factors that cause or may potentially cause or contribute to the occurrence of a defined top event.

## 3. Results

This section contains the analysis of the main emission/exposure events that were reported during the campaign. References are made to the corresponding graph, for each discussed exposure/emission event. As a point of reference for interpreting results, the Short-Term Exposure Limits (STEL) are used. For CPC measurements, the STEL is defined as 80,000 particles/cm^3^ [39], and both far- and near-field exposure values cross over the STEL limit value. Additionally, the current section includes the qualitative FTA to assist the FMEA assessment and aid the identification of the logical connection between the basic events and the corresponding failure modes during the extrusion pilot line.

### 3.1. Airborne Contaminant Exposure

**Figure 3:**
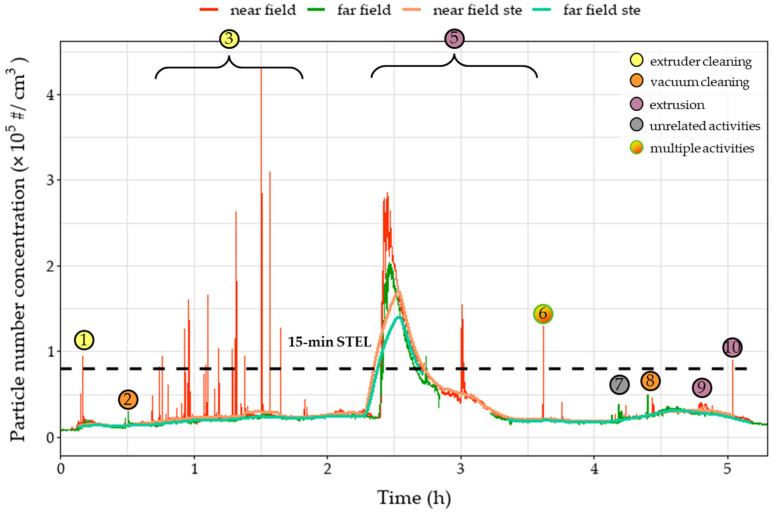
Number concentration of recorded and 15 min average measurements of near- and far-field measurements over time of 10 nm–1 μm particles during the extrusion process of different filaments. Circles denote the time of various events that occurred during the process. Dashed line represents the 15 min Short-Term Exposure Limit (STEL).

**Figure 4:**
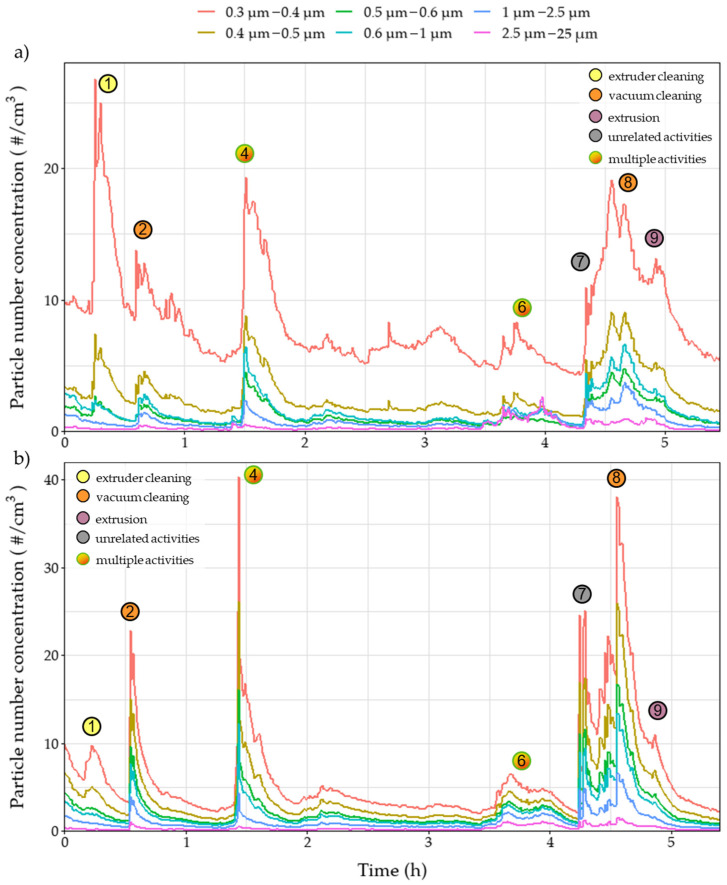
Number concentration of (**a**) near-field and (**b**) far-field measurements over time of 0.3 μm–25 μm particles during the extrusion process of different filaments. Circles denote the time of various events that occurred during the process.

**Figure 5:**
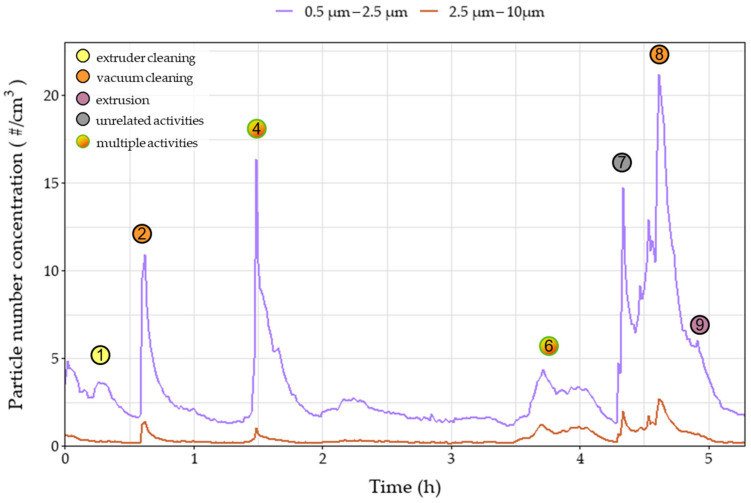
Number concentration of far-field measurements over time of 0.5 μm–10 μm particles during the extrusion process of different filaments. Circles denote the time of various events that occurred during the process.

**Figure 6:**
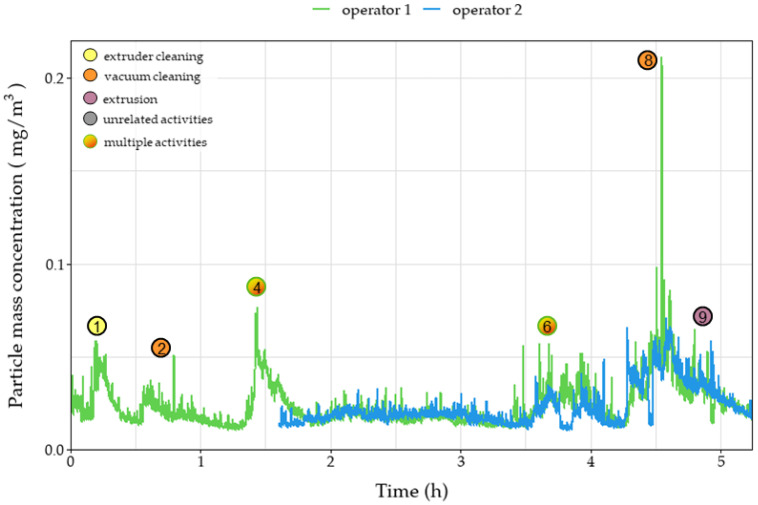
Mass concentration of 0.1 μm–4 μm particle measurements over time, near the breathing zone of the two operators during the extrusion process of different filaments, using the personal monitoring equipment. Lines represent the 10 s moving average of the logged data. Circles denote the time of various events that occurred during the process. Plot of the real-time logged data in Figure S1.

**Figure 7:**
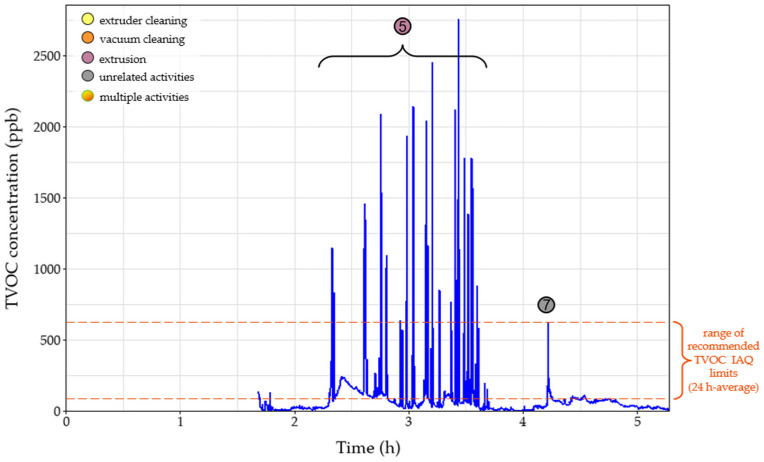
Total volatile organic compounds (TVOC) concentrations of near-field measurements over time during the extrusion process of different filaments. Circles denote the time of various events that occurred during the process. Dashed line represents the 24 h average range of recommended TVOC limits based on Indoor Air Quality (IAQ) Standards.

During the day on which the extrusion process was performed, several emission/exposure events were recorded and connected to various activities that were taking place in the extrusion workroom. Each event is marked on all relevant graphs (Figure 3, Figure 4, Figure 5, Figure 6 and Figure 7). Activities were initiated with the maintenance procedures for proper performance of the following extrusion processes.

Before extruder operation, an extruder cleaning procedure was performed– including scrubbing and cleaning with solvents (dimethylformamide (DMF) and tetrahydrofuran (THF))—resulting in various instantaneous peaks recorded on the number of sub-micron particles as noted in the events one and three (Figure 3). The peaks, reaching values above 400,000 particles/cm^3^, were only observed in the near-field measurements, leading to the remark that these emissions were not dispersed in the far-field sections of the workplace due to the installed ventilation system and the mobile arm hood. Similar trends were observed during event one for the OPC, for particles up to 2.5 μm, and personal monitoring instruments (Figure 4, Figure 5 and Figure 6), reflecting both near- and far-field measurements. It is worth noting that heating the extruder metal mould section for cleaning purposes (removing residue from previous extrusion processes) could also increase the nanoparticle emissions enhancing the magnitude of the observed concentration peaks.

Concentration upsurges were also observed during the use of a vacuum cleaner (event two) mainly for particles larger than 0.3 μm. This finding can be attributed to the limited particle retention properties of the vacuum cleaner filter and the resuspension of previously collected particles. Across all stationary instruments, these peaks were lower in the near-field measurements, since the vacuum cleaner exhaust was facing the far-field measuring instruments, and therefore are not considered a direct result of the extrusion line. Upsurges were more pronounced for particle number concentrations of particles larger than 0.3 μm (Figure 4 and Figure 5) as well as mass concentrations (Figure 6). At 1.5 h (event four), vacuum cleaning was repeated and combined with the manual sieving process (far-field) and extruder part cleaning (near-field). This combination of activities led to peaks similar to those observed during event two.

After the first two hours of cleaning the setup, there was a significant concentration peak for the UFPs both in the near- and far-field measurements (Figure 3) due to the initiation of the extrusion process using pure TPU (event 5). Due to the high concentration values and the long duration of the peak, the 15 min STEL was surpassed for 30 min. Noteworthy, there was no observable increase in the mass concentration measurements in the personal exposure measurements, since the particles emitted were mostly smaller than 300 nm, evidenced by the minor value increases in Figure 4 and Figure 5 and had no impact on the total emitted particulate mass. During event five, several TVOC concentration peaks were observed for the first time for this campaign (Figure 7), exceeding indoor air quality (IAQ) limits shortly but repeatedly. Masterbatch pellets (containing Fe_3_O_4_ NPs) were added to the extrusion hopper at the predefined concentrations (Table 2), after 3.5 h of operation. At this stage, a spike was observed (event six) on the near-field measurements, reaching values of 130,000 #/cm^3^ (Figure 3) for CPC as well as peaks in all channels of Aerotrak (ranging between 3–8 #/cm^3^ for 0.3–20 μm particles) (Figure 4a). The observed peaks in the corresponding far-field Aerotrak readings reached lower concentration values (around and below 5 #/cm^3^). Emissions of larger particles can also be attributed to cleaning and maintenance activities, onset by material failure and disruption of the extrusion process.

A peak in the values of particle number concentrations and TVOC concentration was recorded shortly after the 4 h point (event seven) (Figure 7). This presented a clear increase for the far-field values but was barely, if at all, noticeable for the near-field particle concentration. Furthermore, no adjacent activity was reported for that time; thus, this peak can be attributed to unrelated background activities. Before the completion of the 5 h, the extruded material was changed from TPU to PA, leading to emission event nine. The resulting peaks for particles smaller than 300 nm are contained in the near field with values one order of magnitude lower than those reached during the TPU extrusion. For larger particles, this peak overlaps with the one created by a previous event (event eight), which is attributed to vacuum cleaning. However, particles appear to have spread in the room, reaching the far-field and affecting the values observed by both personal measurement devices. Finally, at the moment of the introduction of the Fe_3_O_4_ NPs/PA masterbatch (event ten), a very short peak is observed on the near-field CPC readings. This is limited to only the smallest particles that do not manage to reach the far-field at this point.

In addition to the near-field/far-field configuration, differences in exposure potential depending on the operator’s position are also displayed through the result of the personal exposure monitoring (Figure 6). During events six and eight, values in the breathing zone of operator 1 are more than triple the concentration in operator 2’s breathing zone (Table 5). This is attributed to each operator’s proximity to the source of the particles since operators were working in different parts of the production line as well as the particle dispersion patterns of the workroom.

While there is a lack of available literature on exposure assessment during polymer extrusion, the potential for emission of UFPs and VOCs during thermal processing of thermoplastics has been documented extensively through studies on fused filament fabrication [40,41,42]. In our previous work [11], the potential for exposure to UFPs has been showcased for 3D printing of polymer matrices similar to the ones in the current study. Sub-micron concentration values were consistent with those presented above, with values quickly increasing at the start of material extrusion and gradually dropping closer to background throughout the process duration. Ding et al. [25] studied the emission patterns during thermal decomposition of polymer filaments and proposed a mechanism for the formulation of particles, based on nucleation of semi-volatile organic compounds (SVOCs).

Regarding the increased concentrations observed during vacuum cleaning, similar results have been demonstrated across the literature [43,44,45]. These studies include measurements of airborne particles in both residential and laboratory settings. Equipping vacuum cleaners with HEPA filters has been shown to greatly reduce emissions [43].

### 3.2. Process Hazard Analysis

**Figure 8:**
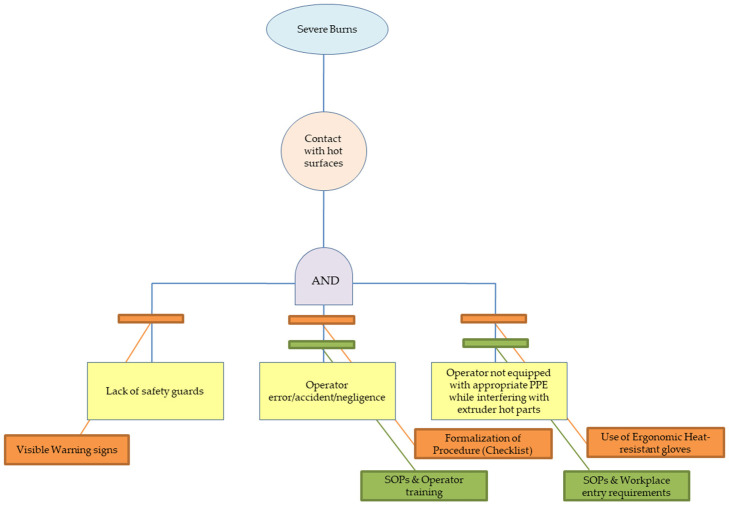
Fault tree leading to severe burns during extrusion (FM1).

**Figure 9:**
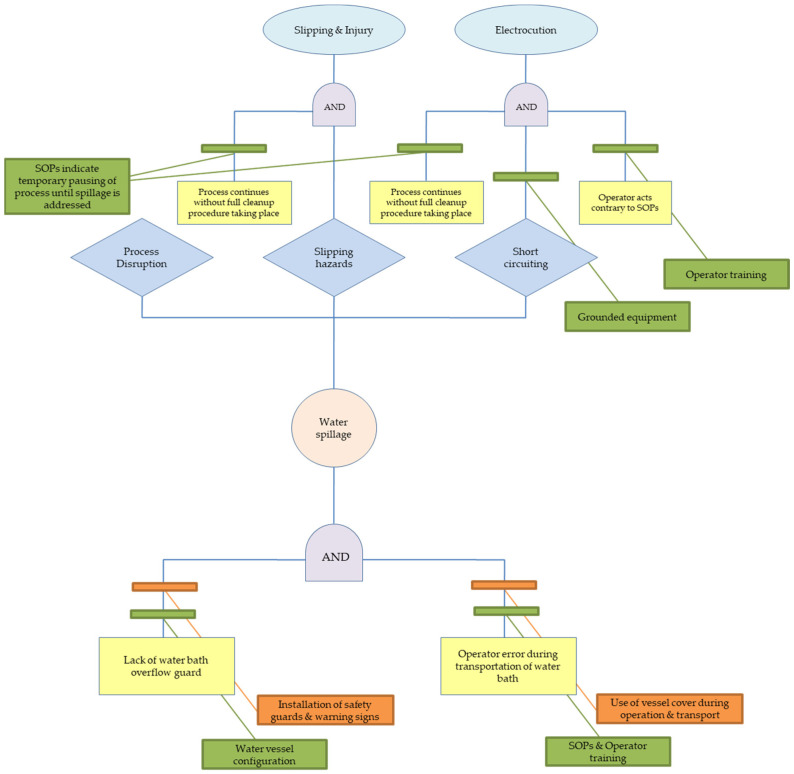
Fault tree leading to electrocution and slipping injury due to water spillage during extrusion (FM2).

**Figure 10:**
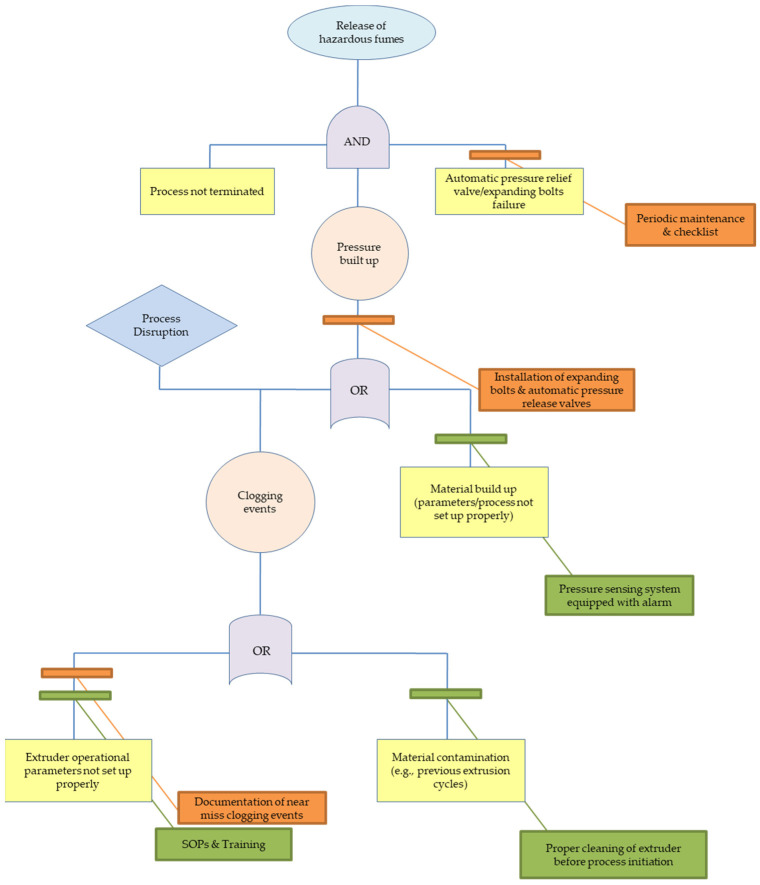
Fault tree leading to clogging and subsequently process disruption and release of hazardous fumes during the extrusion process (FM5).

A total of seven failure modes (FM) were identified by the operators and assigned to the studies nodes. Table 6 presents a short description of each FM, linking the cause and the potential effects. At the same time, there are two different RPN numbers shown in red and green colours. These depict the RPN in the current process controls and the RPN after the use of the recommended actions (which lower the occurrence and detection), respectively. It is worth noting that five FM are linked to the extruder part as the main component of the extrusion pilot line, while one FM is linked to the pelletizer and one FM to the blow dryer. The identified hazards vary from physical hazards (e.g., severe burns, cuts with sharp tooling, high noise levels) to emission hazards (such as misplaced arm hood leading to higher particle concentrations) and events related to process disruption, such as material built-up leading to clogging. The current assessment also includes a qualitative FTA to assist the FMEA assessment and aid the identification of the logical connection between the basic events and the corresponding seven events analysed in Table 6. Additionally, based on the work of Fleury et al. [46], the current (in green colour) and recommended (in orange colour) process controls are included in the FTA trees (Figure 8, Figure 9 and Figure 10)—similar to BowTie analyses—to identify the effectiveness of the safety barriers to mitigate (or eliminate) the risks of the top events.

In detail, FM1 is related to the heating up of the extruder, and during the heating up step at the targeted temperatures (and during extrusion where the equipment is heated up at or above 190 °C), there is a high chance of employees coming in contact with the hot surfaces resulting in severe burns. In this case, the identified causes of the failure mode are the lack of safety guards on the extruder to prevent the operator from coming into contact with the equipment, failure of the current personal protective equipment (PPE) used and the potential operator error during use. The current process safety controls comprise the best practices in place based on the current standard operation procedure, the safety training of lab personnel and the use of PPE required, resulting in a high-risk level (RPN was 32), and remedial actions should be given high priority. A similar set of safety measures, including work instructions and personnel training, are also described in the work of Russkih [47] for the process optimization of extrusion lines.

The recommended actions considered to mitigate the risk were (i) the use of ergonomic heat-resistant gloves to be used throughout the process, which would allow the user to operate the equipment freely as the current heat-resistant PPE in place may discomfort the operator, (ii) lab coats with knit cuffs or other equivalents to be used throughout the task in the extruder, as the lab coat sleeves would prevent the arm from coming in contact with the hot surfaces, and (iii) visible warning signs in multiple positions to be used to alert the operator from coming in contact with the hot surfaces of the equipment. It is worth noting that the use of signs could also lower the risk of operator error. Petretta et al. [48] have reviewed different manufacturing systems and have notified the use of PPE and operating procedures as recommended actions to mitigate the risk of contact with hot surfaces. The recommended set of actions was estimated to lower the occurrence from four to two and the detection from two to one, leading to low-risk levels (the new RPN was eight). In parallel to the FMEA analysis, the FTA evaluation in Figure 8 shows the basic events which can lead to severe burns during extrusion. The “AND” gate denotes three basic events that should take place simultaneously (lack of safety guards, operation error and lack of PPE) for the top event to take place. However, taking into account the current and the recommended controls, the risk is minimized as they can prevent the three events from taking place. 

Additional physical hazards were identified in failure modes FM2, FM3, FM4 and FM6 related to the operation of the extruder and pelletizer instruments. FM2 is related to the water bath where—during the extrusion process—the produced filament passes through water at room temperature to cool down its temperature before the binding step at the spool. However, water spillage is likely as the water tank is exposed due to lack of top cover leading to operator harm (potential electrocution and/or slippery floor leading to slip hazards) as well as equipment hazards such as short-circuiting damaging the equipment resulting in process disruption. The lack of safety guards on the water tank to prevent water spillage and the potential operator error during use has led to an RPN of 30, constituting a high-risk failure, despite the current process controls comprising of the water vessel structure as the water level is below the tank edges, best practices in place and grounding all equipment components, following the existing safety regulations and protocols. Figure 9 shows the series of basic events which can lead to water spillage and subsequently to electrocution and slipping injury. The “AND” gate denotes two basic events that should take place in parallel (lack of safety guards and operation error) for the water spillage to take place. At the same time, a series of parallel events is required (such as spillage negligence and contradiction to SOP) for slipping injury and electrocution to take place. However, taking into account the current and the recommended controls, water spillage can be prevented and associated risks can be significantly minimized leading to a new RPN of five (low-risk level) by installing safety guards on the sides of the water tank to prevent water spillage during use or transport and adding visible warning signs that the water tank is full and avoid any movement/tilting of the tank and lowering the risk of operator’s error. A top cover can also be installed to prevent any water spillage during operation (as filament passes through) and/or during transport of the tank full. All these measures can potentially reduce the likelihood of occurrence and easiness of detectability to their lowest levels (level 1). On the other hand, FM3, FM4 and FM6 that are related to cutting hazards due to unsupervised tooling, head injuries due to misplaced mobile local exhaust ventilation (LEV) unit and cutting injuries from the pelletizer, respectively, have been evaluated at a low-risk level with RPNs varying between four and six, according to our assessment (Table 6). The reason lies in the detectability assigned at one, meaning that these failures can be detected easily; hence, additional control actions focus on minimizing the likelihood of the occurrence via using a toolbelt to hold the tools and placing safety cushions on the LEV unit or even increasing the number of LEV units, minimizing potential misplacement of the existing unit. The FTA assessments of the FM3, FM4 and FM6 are shown in Figures S2–S4 (Supplementary Materials).

The FMEA assessment showed that during the extrusion process, clogging of the twin-screw extruder or other parts of the extrusion is likely to occur either due to material built-up as a result of partial solidification/reduced flowability inside the twin-screw extruder or due to extrusion of large diameter filaments causing errors in the binding process. This can result in high pressure inside the extruder (<300 bar) leading to stall in order to prevent equipment failure; hence, process disruption requiring immediate maintenance action may take place as well as high pressure built-up in the extruder. The identified cause of the failure mode is the contamination of operating materials with previously extruded material of different properties (melting point, glass transition temperature, viscosity, etc.) and inappropriate maintenance. Therefore, current process controls comprise the proper cleaning of the extruder before the extrusion process initiation. Additionally, the prolonged residence times of materials inside the extruder may result in the release of hazardous fumes and airborne hazardous substances (e.g., ultrafine particles and VOCs) as the materials can start decomposing at operating temperatures. Should this happen, it would be reflected in the particle concentrations in Figure 3, leading to elevated number concentrations and probably exceeding the 15 min STE limit, imposing another risk to the operator. Following the current BS EN 1114-1:2011 Standard on the safety requirements for extruders and extrusion lines, automatic pressure release valves equipped with expanding bolts are to be installed. Therefore, above a specific pressure level, the bolt would expand, releasing the excess pressure built-up in the system, preventing the extruder from stalling. Additionally, to mitigate the risk, documentation of the experimental/operation parameters resulting in (potential) clogging events should be recorded; hence, SOPs to be updated and potential clogging events could be identified and prevented. Figure 10 shows the basic events which can lead to clogging of the twin-screw extruder during the extrusion process and potentially the release of hazardous fumes. The “OR” gate denotes either of the two events in series which should take place (either wrong extruder operational parameters or material contamination) for the clogging to take place. However, taking into account the current and the recommended controls, the risk is minimized as they can prevent both events from taking place, lowering the risk level from moderate (RPN: 27) meaning that ‘remedial actions should be taken’ to low levels (RPN: six) and only ‘discretionary remedial actions’ should be considered.

At the same time, another physical hazard is the noise levels during operation due to the compressor of the air blow dryer. As part of the extrusion process, an air blow dryer is installed in series after the water bath for filament drying before binding. However, during the air compression, noise levels exceeded 80 dB which is the lower risk value for a busy workplace. Directive 2003/10/EC [49] describes the harmful effects on the operating personnel when exposed to high environmental noise levels. Additionally, Petretta et al. [48] have described both the physiological and psychophysical stress due to exposure to high noise levels in an occupational environment. Hence, as noise levels are higher than 80 dB for a prolonged amount of time, there is possible hearing damage from continuous noise exposure. The identified cause of the failure mode is the extended operation time of the air compressor for the air blow drying, possibly because of air leakage and the current compressor settings. Despite the current controls, such as best practices in place based on the current standard operating procedure and a pressure sensor on the compressor, additional controls can be considered to mitigate the risk, such as the use of ear protective equipment for prolonged operation time in the extrusion process line (earmuff cancelling noises above a certain limit or the use of single-use earplugs). In that way, operators could be exposed to lower decibel levels minimising the risk level from six to two. The FTA assessment showing the basic events which can lead to hearing damage due to high noise levels during the extrusion process is shown in Figure S5 (Supplementary Materials). 

### 3.3. Study Limitations and Future Research

As part of the OECD “Harmonized Tiered protocol for exposure assessment” [19], a follow-up measurement campaign, including elements of Tier 3 would be applicable to the case presently studied. This is because the Tier 2 measurements, which have been presented in this investigation, have confirmed the emission of nanoparticles and justify proceeding to a more “advanced” assessment. The Tier 3 campaign would involve the collection of workplace air samples and characterisation of the collected particles (e.g., through Scanning Electron Microscope—SEM). By performing this assessment, vital aspects would be clarified, the most critical of which is the possibility of engineered nanomaterial emission in the context of the process. Given that instruments applied in the current campaign do not discern chemical identity, and that the extrusion process itself leads to the emission of nanoparticles as a result of partial degradation of the polymer, it is unclear if the engineered nanomaterials are part of the emissions or not. The fact that specific emission events were connected to nanomaterial handling phases further support that a confirmation of the presence of engineered nanomaterials in the workplace air would be crucial. Additionally, the mode of emission of the engineered nanomaterials would be clarified through the extended assessment. Apart from the possibility that they could be emitted as free particles, the nanomaterials may be emitted in agglomerated formations or may be embedded in polymer matrix particles, as discussed in ISO/TR 22293:2021 [16].

As discussed in the TVOC measurements analysis, the instrumentation limitations lead to difficulties in interpreting the results obtained, since identification of VOC species is not possible. The specific substances emitted could be identified through analytical techniques such as Gas Chromatography–Mass Spectrometry (GC–MS). GC–MS has been consistently and successfully applied to study the VOC emissions within a similar field of study, the FFF 3D printing emission research [42]. It can be argued that, given the complexity and increased cost of GC-MS measurements, a Tiered approach could be recommended in this respect as well. The initial Tier would require the application of a PID sensor, similar to our work, while if emissions are confirmed, a speciation using GC–MS could follow, to further characterize the exposure risk present.

An additional limitation encountered is that the measurements were conducted in one day, with various process phases taking place in sequence (e.g., cleaning processes conducted before the extrusion process start and different materials were introduced sequentially). This led to the processes not being totally isolated from each other and introduced the possibility of the emissions of each phase interfering with the next phases. However, it can be argued that the purpose of such studies is to study exposure potential within a given workplace under typical conditions in terms of workload setup and work intensity. Therefore, since this process workflow is representative of the workplace examined, the study presents value for the exposure assessment under the particular conditions, although showing limitations in terms of how much this information can be generalized. An important point to note is that this limitation is ingrained in the concept of exposure assessment studies in occupational processes since the reproducibility of exact conditions within the work environment is unrealistically challenging in most cases. Waters et al. reviewed occupational exposure assessment methods and argued that since a multitude of factors is in play within the assessment of exposures, variability in different days of measurements is unavoidable. The authors also highlight that while variability cannot be changed, a structured scheme of repeated measurements can assist in defining the levels of variability present and thus support exposure characterization [50]. Therefore, in case the resources for extended Tier 2 measurements are available, these additional repeated measurements can elaborate on the extent of variability present in the results.

Undoubtedly, dedicated and isolated studies on specific materials and processes would be pivotal in clarifying details regarding the emission profiles. Further research can be focused on such “targeted” approaches, such as examining the extrusion of only one material in different process settings. This would assist in understanding the emission potential of the various materials as well as the determinant parameters that may influence the magnitude of emissions for each case (e.g., temperature settings of the extruder, handling method of nanomaterial insertion, etc.).

An important element to note is that an indicative reference exposure threshold was used to interpret the nanoparticle exposure levels. As discussed previously, this does not constitute a health-based limit and is not specific to materials applied in this study. However, despite the uncertainties introduced, it remains a pragmatic approach to understand exposure levels, given the absence of health-based occupational exposure limits for nanoparticles and engineered nanomaterials. Furthermore, given the uncertainty on the presence or not of engineered nanomaterials as discussed previously, it would be invalid to apply a species-specific nanomaterial-based exposure threshold.

Regarding the process safety part of the study, it should be noted that the data for the FMEA and FTA examinations were obtained through bilateral interviews with the process operators, and no prior event occurrence data was available. Documentation of near-miss events or accidents that may have occurred in the context of the nanocomposite manufacturing line across the spectrum of its operation (e.g., yearly reports) could have supported the assessment with historical data, in the sense that some of the events may have occurred in the past, indicating a confirmation of their occurrence probability. Such an endeavour was presented in a study by Gopalaswami et al. [51], wherein the authors developed a laboratory incident database for laboratories handling hazardous chemicals, the majority of which were located in universities. A basic collection of information on each incident was undertaken, through data sources such as incident reports or institutional databases, also documenting the cause and consequences of the events. The events were categorized into incident types (e.g., explosions, chemical exposures, etc.), while the substances, consequences and causes were also classified. Through this approach, the authors managed to identify the most frequently recurring hazardous events in the laboratory, the most prevalent of which were chemical spills, explosions and fires, while interestingly, it was found that the most common incident cause was improper storage and handling. Given the newness of the nanocomposite manufacturing technologies and the still-developing experience and knowledgebase, collection of deviation, hazardous incident and near-miss data would be quite beneficial for the further development of this sector and would significantly support the performance of hazard analysis studies.

## 4. Conclusions

This study presents an extended methodology for the evaluation of occupational risk and safety, applied for a nanocomposite production line. Measurements of airborne contaminant concentrations were coupled with process hazard approaches (FMEA and FTA). Measurements showed an increased exposure potential at the start of the extrusion process, consistent with what is reported in the literature for similar processes. Additionally, emissions of particles were observed during the cleaning activities. Seven failure modes were identified and were further analysed through FTA. The currently applied control systems were documented, and additional mitigation actions were proposed. The estimated RPN after the implementation of the suggested controls provides an understanding of their impact and enables the prioritization of the most effective mitigation measures. Further studies on other recycling processes can provide a wider understanding of the risk and safety aspects and allow for more effective implementation of a control system in the early stages of process design.

## Figures and Tables

**Table 1:** Workroom and process characteristics.

Process Characteristics	Logged Specification
Workroom Ventilation/Air filtration	Mechanical dilution ventilation, arm hood above the extruder
Workroom Specifications	Volume: ~250 m^3^ Temperature: ~25 °C Humidity: ~40%
Process Duration	4 h to 8 h workday
Operator Involvement	2 to 3 operators

**Table 2:** Position, type and measurement size range of instruments used in the exposure assessment campaign).

Position	Equipment Name	Measurement
Near-field	CPC 3007, TSI Inc.	10 nm–1 μm (number particle concentration)
	Aerotrak 9306-V2, TSI Inc.	0.3 μm–25 μm (number particle concentration)
	Tiger, Ionscience	0–20,000 ppm (isobutylene equivalent)
Far-field	CPC 3007, TSI Inc.	10 nm–1 μm (number particle concentration)
	Aerotrak 9306-V2, TSI Inc.	0.3 μm–25 μm (number particle concentration)
	DC1700, Dylos	0.5 μm–10 μm (number particle concentration)
Portable	SidePak AM520i, TSI Inc.	0.1 μm–4 μm (mass-based concentration)
	SidePak AM520i, TSI Inc.	0.1 μm–4 μm (mass-based concentration)

**Table 3:** Level description per failure mode and effect analysis (FMEA) factors.

Level	Severity (S)	Occurrence (O)	Detection (D)
1	Negligible	Unlikely	Almost certain
2	Minor	Seldom	High
3	Serious	Occasional	Moderate
4	Major	Likely	Low
5	Fatal	Frequent	Almost impossible

**Table 4:** Risk scoring table for 5 × 5 × 5 matrix of the FMEA analysis.

Risk Level	Min	Max	Description
Very low	0	1	Negligible associated risks
Low	2	8	Discretionary remedial actions
Moderate	9	27	Remedial actions should be taken
High	28	64	Remedial actions must be given high priority
Very high	65	125	Operation not permissible. Immediate actions necessary

**Table 5:** Summary table of emission/exposure events and peak concentrations.

	Emission Events	1 Extruder Cleaning	2 Vacuum Cleaning	3 Extruder Cleaning	4 Multiple Activities	5 Extrusion	6 Multiple Activities	7 Unrelated Activities	8 Vacuum Cleaning	9 Extrusion	10 Extrusion
Instruments											
CPC 1 (#/cm^3^)		95,000	\	431,000	\	286,000	130,000	37,300	46,100	39,100	90,400
CPC 2 (#/cm^3^)		20,800	\	30,500	\	204,000	\	37,800	49,500	\	\
Aerotrak 1 (#/cm^3^)	Ch1	20.645	13.763	\	19.305	\	8.319	13.151	19.118	12.609	\
	Ch2	5.314	4.546	\	8.777	\	2.980	5.588	9.097	4.955	\
	Ch3	1.873	2.093	\	4.527	\	1.354	3.178	4.803	2.539	\
	Ch4	1.746	2.850	\	6.442	\	1.846	4.423	6.667	2.934	\
	Ch5	0.605	1.488	\	2.399	\	2.268	2.517	3.731	1.309	\
	Ch6	0.209	0.442	\	0.513	\	2.614	0.972	0.943	0.584	\
Aerotrak 2 (#/cm^3^)	Ch1	9.249	22.789	\	40.331	\	6.524	25.105	38.023	11.054	\
	Ch2	4.741	15.059	\	26.133	\	4.600	17.457	25.900	7.020	\
	Ch3	2.498	9.655	\	16.076	\	3.383	11.667	16.732	4.257	\
	Ch4	1.873	7.692	\	12.209	\	2.955	9.465	13.409	3.319	\
	Ch5	0.835	4.008	\	4.380	\	2.090	4.879	6.586	1.562	\
	Ch6	0.214	1.044	\	0.600	\	1.045	1.281	1.541	0.558	\
Dylos (#/cm^3^)	Ch1	3.613	10.944	\	5.025	\	4.361	14.737	21.196	6.124	\
	Ch2	0.321	1.413	\	0.456	\	1.278	2.013	2.687	0.805	\
SidePak 1 (mg/m^3^)		0.135	0.081	\	0.277	\	0.281	\	0.678	0.211	\
SidePak 2 (mg/m^3^)		\	\	\	\	\	0.085	\	0.185	0.240	\
Tiger (ppb)		\	\	\	\	2759	\	622	\	\	\

“\” Indicates that no significant deviations from the background concentrations were displayed during this event for the instrument/size channel described. Red colour indicates surpassing of exposure limit (STEL), and orange colour indicates high values.

**Table 6:** FMEA analysis. Red and green colours denote the Severity (S), Occurrence (O), Detectability (D) and Risk Priority Numbers (RPN).

#	Equipment	Process Step	Potential Failure Mode	Potential Failure Effect	S	Potential Causes of Failure	O	Current Process Controls	D	RPN	Recommended Actions	S	O	D	RPN
FM1	Extruder	Heating up of extruder	Contact with hot surfaces	Severe burns	4	Lack of safety guards, PPE not applied, operator error	4	Best practices, personnel training, PPE required	2	32	Ergonomic heat-resistant gloves, lab coat and visible warning signs, formalization of procedure (checklist)	4	2	1	8
FM2	Extruder	Extruder water bath	Water spillage	Electrocution, short-circuiting and spillage causing slippery floor leading to slip hazards, process disruption	5	Lack of safety guards, operator error	2	Water vessel configuration, best practices and grounded equipment	3	30	Installation of safety guards and warning signs, vessel cover during operation and transport	5	1	1	5
FM3	Extruder	Maintenance tooling	Sharp pointy tools throughout the workplace	Cutting hazards, injuries	3	Lack of appropriate case/tool storage, operator error	2	Best practices, operator training, PPE required	1	6	Update SOP, use of tool belt for frequently used tooling, use of designated toolkit for positioning unused tools	3	1	1	3
FM4	Extruder	Positioning of the LEV	Misplaced arm hood	Physical hazards—head injury	2	Continuous repositioning of LEV to points of high emissions, operator error	2	Best practices and SOPs	1	4	Installation of multiple LEVs to avoid misplacement, cushion covers on arm hood external surfaces/edges	2	1	1	2
FM5	Extruder	Extrusion	Clogging	Release of hazardous fumes, contact with heated material during cleaning, high pressure built-up.	3	Contamination (e.g., previously extruded material)	3	Proper cleaning of extruder before process initiation, pressure-sensing system, pressure alarm	3	27	Documentation of near-miss clogging events that could lead to prediction of clogging events through process monitoring. Pressure-release valves.	3	2	1	6
FM6	Pelletizer	Pelletizer	Mechanical parts exposed	Injuries and cutting	5	Safety door opened during operation, operator error	1	Best practices and safety door, alarm	1	5	Alarm to notify that the door is open and interlock to prevent pelletizer to start when door is open. Periodic maintenance	5	1	1	5
FM7	Blow dryer	Drying	High noise levels (>80 dB)	Possible hearing damage from continuous exposure	2	Extended operation of air compressor (leakage, compressor settings)	3	Best practices, process monitoring (pressure sensors)	1	6	Earmuffs/Earplugs	2	1	1	2

## Data Availability

Data is contained within the article and Supplementary Materials.

## Supplementary Materials

The following supporting information can be downloaded at: https://www.mdpi.com/article/10.3390/polym15132836/s1. Figure S1: Recorded data of the mass concentration of 0.1 μm–4 μm particle measurements over time, near the breathing zone of the two operators during the extrusion process of different filaments, using the personal monitoring equipment. Circles denote the time of various events that occurred during the process; Figure S2: Fault tree leading to cutting during the extrusion process (FM3); Figure S3: Fault tree leading to head injury due to LEV misplacement during the extrusion process (FM4); Figure S4: Fault tree leading to cutting injury during pelletizing (FM6); Figure S5: Fault tree leading to hearing damage during extrusion (FM7).
