# Supplementary material for: Integrating Exposure Assessment and Process Hazard Analysis: The Nano-Enabled 3D Printing Filament Extrusion Case
Source: Polymers (Basel). 2023 Jun 27;15(13):2836. doi: 10.3390/polym15132836 (PMC10346662; doi:10.3390/polym15132836)
Supplement: Supplementary file 1 [file polymers-15-02836-s001.zip › polymers-2447664-supplementary.pdf]

Supplementary Materials

Integrating exposure assessment and process hazard analysis: The nano-enabled 3D printing filament extrusion case

Stratos Saliakas[1], Spyridon Damilos[1], Melpo Karamitrou[2], Aikaterini-Flora Trompeta[2], Tatjana Kosanovic Milickovic[2], Costas Charitidis[2] and Elias P. Koumoulos[1]*

[1] Innovation in Research & Engineering Solutions (IRES), 1780 Wemmel, Belgium

[2] Research Lab of Advanced, Composites, Nanomaterials and Nanotechnology (R-NanoLab), School of Chemical Engineering, National Technical University of Athens, Zographos, 15780 Athens, Greece

* Corresponding author: epk@innovation-res.eu

Contents

S1. Airborne particle measurement concentrations 2

S2. Fault tree analysis (FTA) of fFailure mModes.. 3

S1. Airborne particle measurement concentrations

Figure S1~~Figure S1~~ presents the values of particle mass concentration as they were recorded using~~by~~ SidePak AM520i, in 1 second logging intervals. Several emission events, denoted with coloured circles, are observed. All emission events are~~ being~~ discussed in detail in the main text.

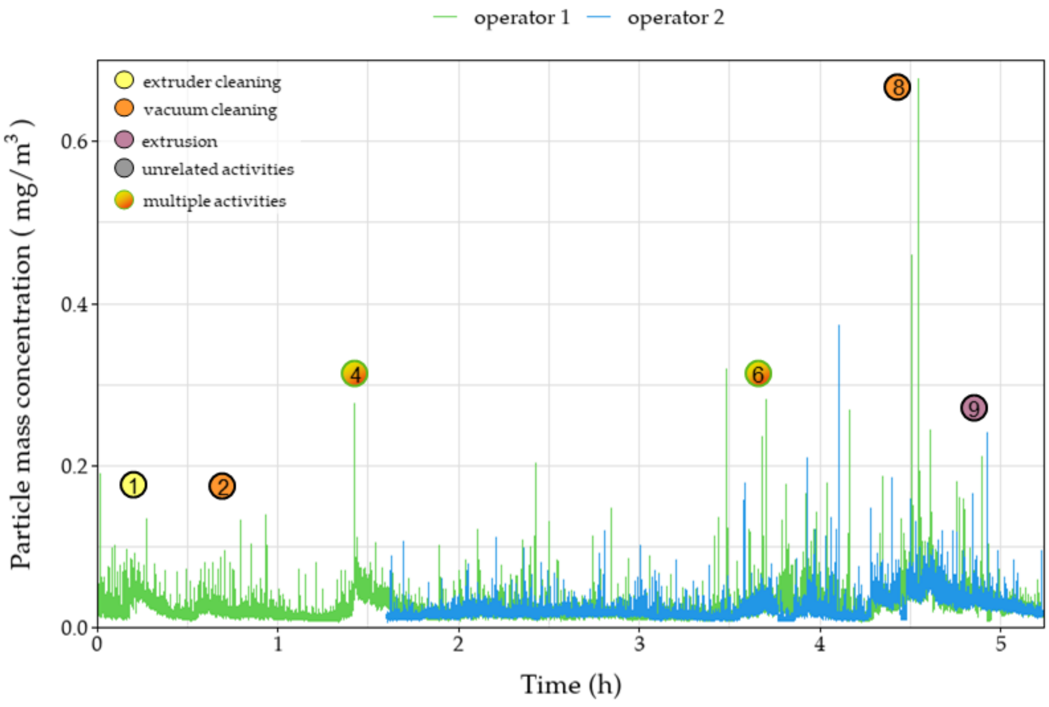

Figure S1. *Recorded data of the mass concentration of 0.1 µm~~—~~4 µm particle measurements over time, near the breathing zone of the two operators during the extrusion process of different filaments, using the personal monitoring equipment. Circles denote the time of various events that occurred during the process.*

S2. Fault tree analysis (FTA) of f~~F~~ailure m~~M~~odes

Failure Mode #1 (Heating up of extruder)

During heating up of the extruder at the targeted temperatures (and during extrusion where the equipment is heated up at/above 190 ^{o}C), there is a high chance of employees coming in contact with the hot surfaces resulting in severe burns. The identified causes of the failure mode are the lack of safety guards on the extruder to prevent the operator from coming into contact with the equipment, failure of the current personal protective equipment (PPE) applied and the potential operator error during use. The current process controls comprise~~ of~~ the safety best practices in place based on the current standard operation procedure, the safety training of lab personnel and the use of PPE required.

The current RPN is~~:~~ RPN = S x O x D = 4 x 4 x 2 = 32.

Recommended actions in order to mitigate the risk include first of all the use of ergonomic heat-~~-~~resistant gloves to be used throughout the process, which would allow ~~to ~~the user to operate the equipment freely as the current heat-resistant PPE in place may discomfort the operator. Lab coat (lab coats with knit cuffs or other equivalents) should be used throughout the task in the extruder~~,~~ as the lab coat sleeves would prevent the arm from coming in contact with the hot surfaces. Visible warning signs should be positioned in multiple places to alert the operator from coming in contact with the hot surfaces of the equipment. Use of signs could also lower the risk of operator's error.

The new RPN would be~~:~~ RPN = S x O x D = 4 x 2 x 1 = 8.

Failure Mode #2 (Extruder water bath)

During the extrusion process, the produced filament passes through a water bath to cool down the filament temperature before the binding step at the spool. However, water spillage is likely as the water tank is exposed due to lack of top cover leading to operator harm (potential electrocution and/or slippery floor leading to slip hazards)~~,~~ as well as equipment hazards such as short-circuiting damage of~~ing~~ the equipment resulting in process disruption. The identified causes of the failure mode are the lack of safety guards on the water tank to prevent the water spillage and the potential operator error during use. The current process controls comprise~~ of~~ the water vessel structure as the water level is below the tank edges, safety best practices in place based on the current standard operation procedure and all equipment in the laboratory is grounded following the existing safety regulations and protocols.

The current RPN is~~:~~ RPN = S x O x D = 5 x 2 x 3 = 30.

In order to mitigate the risk, safety guards should be installed on the sides of the water tank in order to prevent water spillage during use or transport. Additionally, visible warning signs signalling that the water tank is full and avoid any movement/tilting of the tank would mitigate the associated risk. The sign could also lower the risk of operator's error. A top cover

should be installed in order to prevent any water spillage during operation (as filament passes through) and/or during transport of the tank full.

The new RPN would be~~:~~ RPN = S x O x D = 5 x 1 x 1 = 5.

Failure Mode #3 (Maintenance tooling)

During the cleaning and maintenance of the extruder (and the equipment associated with the process line in general), the required tools are placed on top of the workbench. Hence, sharp and pointy tools are placed on the workplace benches, ~~and~~ resulting in potential cutting hazards and injuries. The identified causes of the failure mode are the lack of appropriate case/tool storage and the potential operator error during maintenance. The current process controls comprise ~~of~~ the safety best practices in place based on the current standard operation procedure, the safety training of lab personnel and the use of PPE required.

The current RPN is~~:~~ RPN = S x O x D = 3 x 2 x 1 = 6.

In order to mitigate the risk, the current standard operating procedures (SOP) could be amended in order to include the best practices, event series and storage options for the required tools. In that way, likelihood of tools being scattered on the workbench will be substantially lower. A tool belt could be used by the operator(s) in order to place (and store) the frequently used tools during operation and maintenance and subsequently minimize the number of tools on the workbench, while a designated toolkit (or toolbox) could be used for positioning the unused tools during maintenance.

The new RPN would be~~:~~ RPN = S x O x D = 3 x 1 x 1 = 3.

Figure S2~~**Figure S2**~~ shows the basic events which can lead to cutting during the extrusion process. The “AND” gate denotes three basic events should take place in parallel (inappropriate storage, mishandling and lack of PPE) in order for the top event to take place. However, taking into account the current and the recommended controls, risk is minimized as they can prevent the three events from taking place.

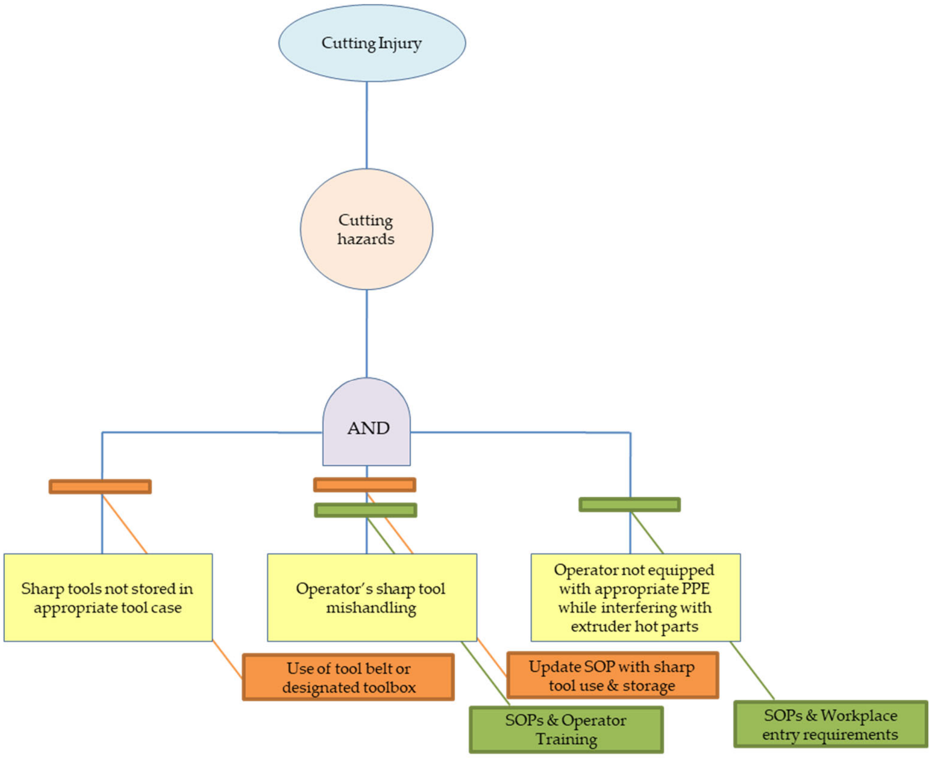

Figure S2. *Fault tree leading to cutting during the extrusion process (FM3).*

Failure Mode #4 (Positioning of the LEV)

During the extrusion process, the local exhaust ventilation unit (LEV) in place (arm hood) is being constantly repositioned to positions of high emission potential or in order to make up space for the operator when user's interference is required during operation or maintenance. Hence, physical hazards such as head injury is highly likely by misplacing the arm hood in the operator's proximity zone. The identified causes of the failure mode are the continuous reposition of LEV to points of high emissions and the potential operator error during use. The current process controls comprise ~~of~~ the safety best practices in place based on the current standard operation procedure.

The current RPN is~~:~~ RPN = S x O x D = 2 x 2 x 1 = 4.

In order to mitigate the risk, additional LEV units could be installed on the extrusion process line~~,~~ in order to minimize the continuous repositioning of the arm hood and subsequently mitigating the risk of head injury due to the misplacement, while also adding an additional layer of protection for process emission hazards. Use of cushion cover on the arm hood external surfaces and edges could ~~will~~ mitigate substantially the risk of head injury and any other physical hazard by the LEV unit.

The new RPN would be~~:~~ RPN = S x O x D = 2 x 1 x 1 = 2.

[Figure S3](#)~~Figure S3~~ shows the basic events which can lead to head injury during the extrusion process. The "AND" gate denotes two basic events should take place in parallel (LEV reposition and operator's error) in order for the top event to take place. However, taking into account the current and the recommended controls, risk is minimized as they can prevent the two events from taking place.

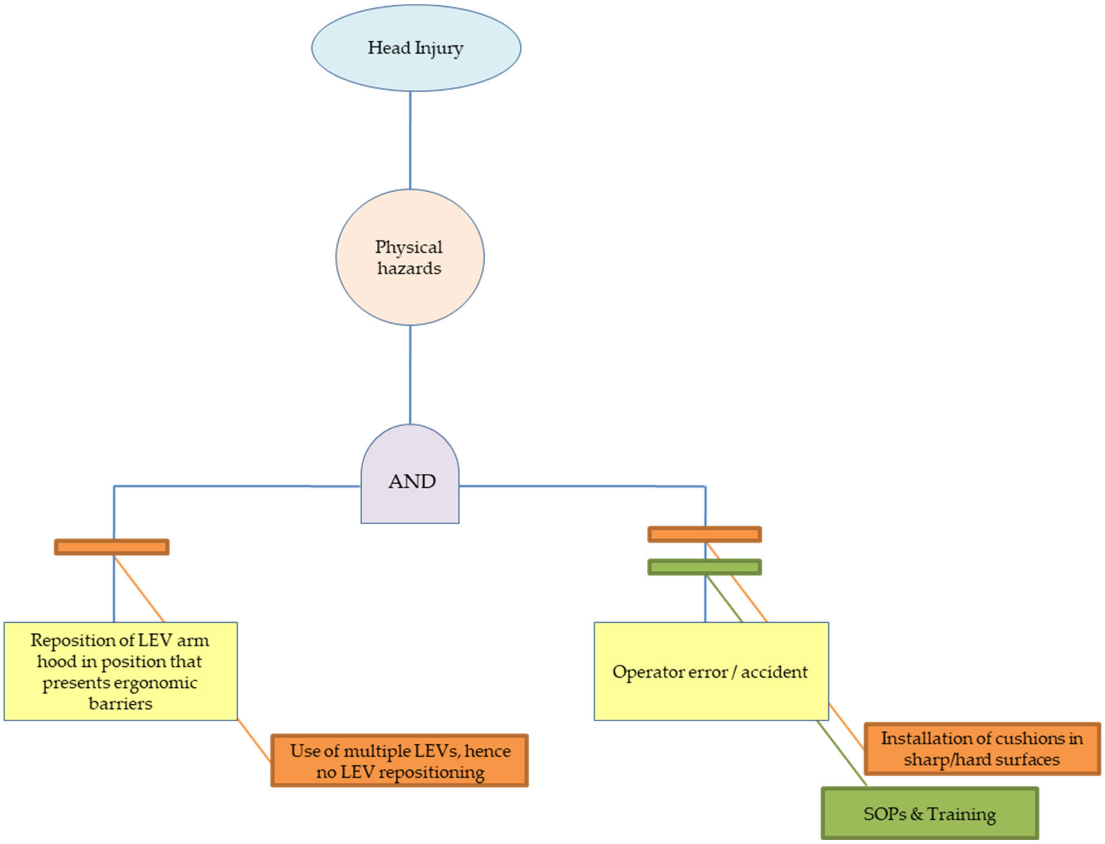

Figure S3. *Fault tree leading to head injury due to LEV misplacement during the extrusion process (FM4).*

Failure Mode #5 (Extrusion~~-~~—Clogging)

During the extrusion process, clogging of the twin-screw extruder or other parts of the extrusion is likely to occur either due to material partial solidification/reduced flowability inside the twin-screw extruder or due to extrusion of large diameter filaments causing errors in the binding process~~,~~ as well as release of hazardous fumes due to the prolonged residence times at the elevated temperatures. Hence, process disruption requiring immediate maintenance action is needed as well as high pressure built-up in the extruder. The identified cause of the failure mode is the contamination of operating materials with previous extruded material of different properties (melting point, glass transition temperature, viscosity, etc.). The current process controls comprise~~ of~~ the proper cleaning of extruder before extrusion

process initiation as well as a pressure-sensing system connected to the extruder software equipped with an alarm, which goes off above a certain pressure threshold.

The current RPN is RPN = S x O x D = 3 x 3 x 3 = 27.

In order to mitigate the risk, documentation of the experimental/operation parameters resulting in (potential) clogging events should be considered. In that way, SOPs to be updated and potential clogging events could be identified and prevented. Following the current BS EN 1114-1:2011 Standard on the safety requirements for extruders and extrusion lines, automatic pressure release valves equipped with expanding bolts to be installed. Therefore, above a specific pressure level, bolt would expand, releasing the excess pressure built-up in the system.

The new RPN would be RPN = S x O x D = 3 x 2 x 1 = 6.

Failure Mode #6 (Pelletizer)

As part of the extrusion process, pellets in the hopper are cut into small pieces (< 4mm) using an enclosed pelletizer. However, the enclosure opens during the collection of the plastic pellets, and the jagged mechanical parts are exposed. This could potentially lead to operator injury. The identified causes of the failure mode are ability to open the safety door of the enclosure during operation and the potential operator error during use. The current process controls comprise ~~of~~ the safety best practices in place based on the current standard operation procedure and the safety door of the enclosure equipped with an alarm signalling when the door is open, exposing the mechanical parts of the pelletizer.

The current RPN is RPN = S x O x D = 5 x 1 x 1 = 5.

In order to mitigate the risk, an interlock should be installed connecting the door with the operating butting. Hence, it would prevent the pelletizer from starting when door is open or immediately shutting off the equipment if the door accidentally opens during operation.

The new RPN would be RPN = S x O x D = 5 x 1 x 1 = 5.

Figure S4~~Figure S4~~ shows the basic events which can lead to severe cutting from the pelletizer. The "AND" gate denotes three basic events should take place in parallel (opening the safety door during operation, operator's error and malfunction of the alarm system to signal that the safety door is open) in order for the top event to take place. However, taking into account the current and the recommended controls, risk is minimized as they can prevent the three events from taking place.

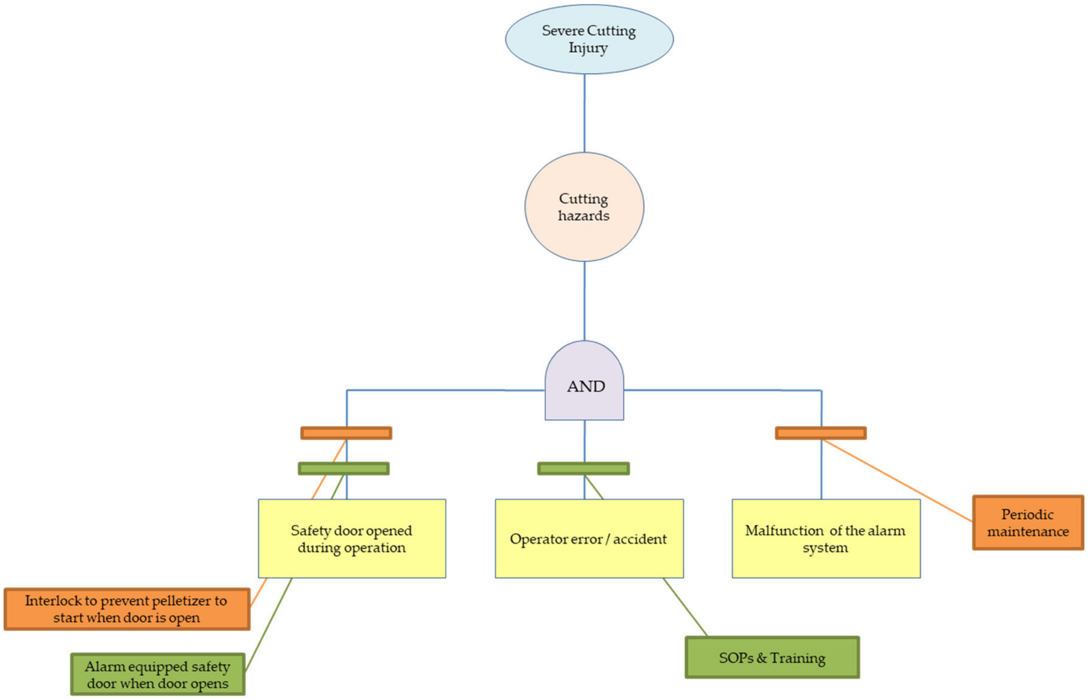

Figure S4. *Fault tree leading to cutting injury during pelletizing (FM6).*

Failure Mode #7 (Blow dryer)

The identified cause of the failure mode is the extended operation time of the air compressor for the air blow drying, possible because of air leakage and the current compressor settings. The current process controls comprise of the safety best practices in place based on the current standard operation procedure and a pressure sensor for process monitoring.

The current RPN is: RPN = S x O x D = 2 x 3 x 1 = 6.

In order to mitigate the risk, operators should wear ear protective equipment for prolong operation time in the extrusion process line. The protective equipment could be either earmuff cancelling noises above a certain limit or the use of single-use earplugs.

Hence, the new RPN would be: RPN = S x O x D = 2 x 1 x 1 = 2.

Figure S5Figure S5 shows the basic events which can lead to hearing damage due to high noise levels during the extrusion process. The "OR" gate denotes either of the two events in series which should take place (extended operation of the air compressor due to leakage or faulty compressor settings) in order for noise levels to surpass the critical limit. However, taking into account the current and the recommended controls, risk is minimized as they can prevent both the two events from taking place.

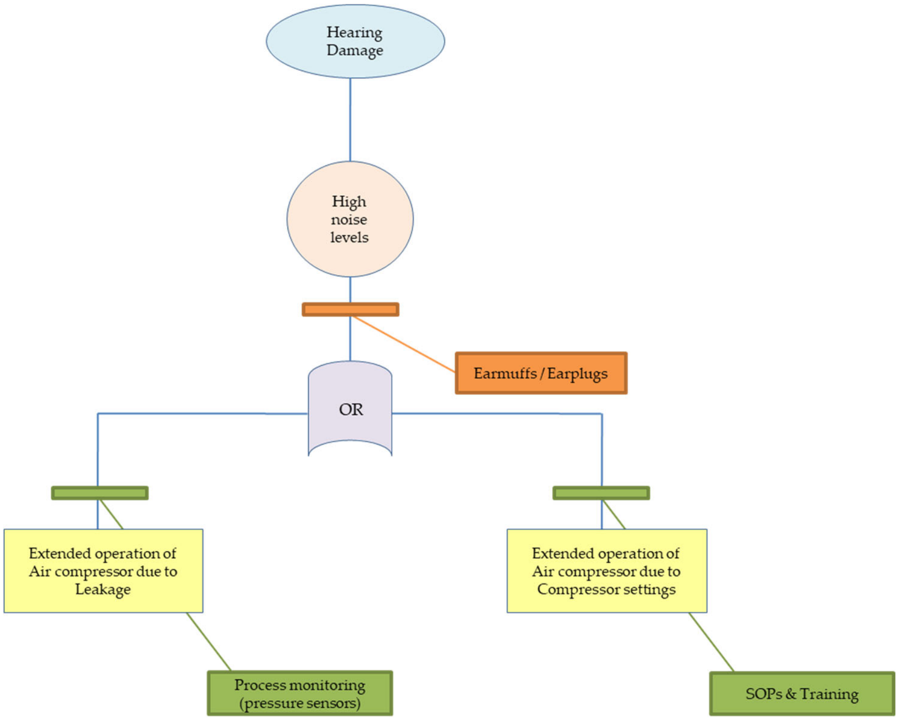

Figure S5. *Fault tree Fault tree leading to hearing damage during extrusion (FM7).*
